# ALCAPA Presents in an Adult with Exercise Intolerance but Preserved Cardiac Function

**DOI:** 10.1155/2012/471759

**Published:** 2012-08-29

**Authors:** Yan Liu, Beth W. Miller

**Affiliations:** Department of Internal Medicine, University of Texas Southwestern Medical School Residency Programs at Austin, University Medical Center Brackenridge, 601 E 15th Street, Austin, TX 78701, USA

## Abstract

Anomalous origin of the left coronary artery from the pulmonary artery (ALCAPA) is a rare congenital anomaly that usually manifests as severe left-sided heart failure and mitral valve insufficiency during the first one to two months of life. The majority of these cases die in infancy if not corrected early upon presentation. Adulthood presentation is rare and most of the untreated patients who reach adulthood present with left ventricular dysfunction, severe mitral regurgitation, and sometimes myocardial infarction. Here we report a case of a 20-year-old woman with a history of exercise intolerance since childhood that was misinterpreted as asthma until a 2D-Echo revealed ALCAPA with RCA collaterals to the left anterior descending artery, preserved LV ejection fraction, and absence of apparent mitral valve abnormality. One month after the ALCAPA diagnosis, she successfully underwent surgical reconstruction of left main and pulmonary artery without any major complications. She had normal left ventricular function without apparent ischemic cardiac symptoms eighteen months after procedure.

## 1. Introduction

Anomalous origin of the left coronary artery from the pulmonary artery (ALCAPA) is a rare congenital anomaly occurring in 1 of 300,000 live births [1, 2] and accounts for 0.4 percent of congenital cardiac abnormalities. ALCAPA was first reported in 1885 by Brooks [1] and its first clinical description in conjunction with autopsy findings was described by Bland et al. in 1933 [3]. Therefore the condition is also known as Bland-White-Garland-syndrome. 

The ALCAPA anomaly may result from abnormal septation of the conotruncus into the aorta and pulmonary artery, or from persistence of the pulmonary buds together with involution of the aortic buds that eventually form the coronary arteries. ALCAPA is usually an isolated cardiac anomaly that does not present prenatally because of the favorable fetal physiology that includes equivalent pressures in the main pulmonary artery and aorta secondary to a nonrestrictive patent ductus arteriosus, and the relatively equivalent oxygen concentrations due to parallel circulations. This results in normal myocardial perfusion and, therefore, no stimulus for collateral vessel formation between the right and left coronary artery systems is present. Shortly after birth, as the circulation becomes one in series, pulmonary artery pressure and resistance decrease, as does oxygen content of pulmonary blood flow. This causes a drop in antegrade flow and oxygen content of the anomalous left coronary artery, leading to myocardial ischemia. Furthermore, collateral circulation between the right and left coronary systems ensues and the left coronary artery flow reverses and enters the pulmonary trunk due to the low pulmonary arterial pressure, causing coronary steal phenomena. Consequently, fixed ischemia or myocardial infarction occurs. Eighty-five percent of all cases of ALCAPA present within the first two months of life. The typical clinical course involves severe left-sided heart failure and significant mitral valve insufficiency secondary to papillary muscle ischemia, permanent fibrosis, or left ventricular dilatation. The late presentation is extremely rare and usually with significant cardiac function compromise.

## 2. Case Presentation

**Figure 1:**
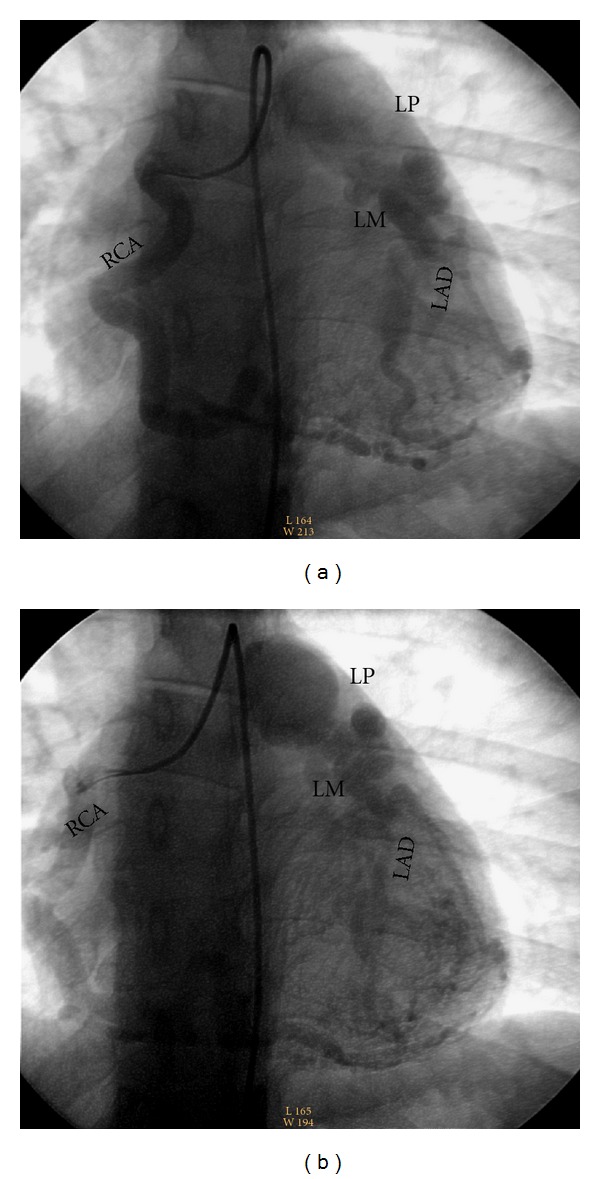
Coronary angiogram showing ALCAPA with left-to-right shunt. Retrograde filling was seen through collaterals arising from the enlarged right coronary artery (RCA), which gives rise to collaterals to a large left anterior descending artery (LAD) and left main artery (LMA) originated from the left main pulmonary (LP) trunk. The LAD and LMA are filling the pulmonary artery through a pronounced left-to-right shunt.

A 20-year-old woman presents with chronic dyspnea on exertion and exercise intolerance that was attributed to and treated as presumed exercise-induced asthma since childhood. She experienced worsening left-sided chest heaviness and was subsequently referred to our institute. She has no coronary risk factors and no family history of premature coronary artery disease or congenital heart condition. Physical examination was normal except a subtle continuous murmur at apex and lower right sternal border. ECG showed sinus rhythm without ST or T wave changes (Supplemental Figure 1 available at doi:10.1155/2012/471759). Chest X-ray showed no cardiomegaly (Supplemental Figure 2). Serial cardiac enzymes were negative. In the light of cardiac murmur, an echocardiogram was obtained and revealed anomalous coronary arteries with a large left coronary artery to main pulmonary artery fistula. There was mild left ventricular enlargement with preserved left ventricular contractile function and an ejection fraction of 65%. The appearance of the left ventricle and left atrium was consistent with a systemic to pulmonary vascular shunt with increased stroke volumes in the left heart. There were no structural abnormalities of the aortic, mitral, tricuspid, or pulmonic valves, and there was very mild mitral regurgitation by Doppler. A subsequent cardiac catheterization confirmed the diagnosis of ALCAPA with retrograde filling through collaterals arising from an enlarged right coronary artery (12 mm) (Figure 1), pronounced left-to-right shunting from the left main coronary artery into the left main pulmonary artery trunk; and the right coronary artery gives rise to collaterals to a large left anterior descending artery which has ectasia in its proximal segment into a smaller circumflex artery. The patient underwent surgical treatment by the creation of a pulmonary artery tube graft from the aorta to the left coronary artery and reconstruction of the main pulmonary artery with a bovine pericardial patch. She had no major complications or ischemic symptoms 18 months after operation. A follow-up echocardiogram at that time showed normalized stroke volume and left atrial and ventricular size secondary to reversed left-to-right shunt, preserved LV EF at 55%, and absence of valvular abnormalities. 

## 3. Discussion

As a rare congenital heart condition, ALCAPA even more rarely has a late presentation in adulthood as few of these patients survive past childhood without surgical repair [4]. Among the reported adult cases [5–10], most of them, if not all, presented with evidence of irreversible impairment of cardiac function such as severe dilated cardiomyopathy, sudden cardiac death, acute myocardial infarction, malignant arrhythmias secondary to myocardial scar tissue, impaired LV contractile function, and development of significant mitral regurgitation. In this case, although she has an adulthood presentation, her presentation with a preserved LV contractile function without apparent structural or functional cardiac impairment may have prevented her from a life-threatening event later in life. While it is known that the sufficient collateral blood supply from the right coronary artery could help some patients with ALCAPA pass through childhood with relatively minor symptoms, such as dyspnea, chest pain, and exercise intolerance, these symptoms are often misinterpreted, (as it was initially the case for the presented patient), as exercise-induced asthma. The key for an early diagnosis before the occurrence of permanent cardiac damage is a careful cardiac exam and detailed physical examination, maintaining a high clinical suspicion when children have exercise intolerance. Early employment of first line diagnostic modality echocardiogram is essential, as ECG in this case is usually unrevealing. 

In infants, most of the patients with surgically corrected ALCAPA show normalization of both ventricular function and mitral valve insufficiency [11, 12]. Estimated long-term survival at 20 years was recently shown to be 94.8% [12]. Although there is no large clinical trial study on surgically corrected adult ALCAPA, the prognosis is usually determined by the extent of irreversible left ventricular dysfunction and presence of myocardial scar tissue. This patient had almost complete reversal of previous left atrial and ventricular dilation with maintained normal LV contractile function 18 months after surgical repair, which is largely attributed to the absence of irreversible LV dysfunction or myocardial infarction when the diagnosis was made.

In conclusion, even as a rare scenario of a rare disorder, diagnosis of adulthood ALCAPA should be considered not only in adult patients presenting with clear evidence of ischemic heart disease, left ventricular dysfunction, or arrhythmias, but also more importantly in patients with minor symptoms of exercise intolerance or dyspnea that could be easily misinterpreted or missed without high clinical suspicion and detailed physical and diagnostic evaluation, as early diagnosis and timely surgical treatment usually results in an excellent prognosis.

## Supplementary Material

Supplemental Figure 1: 12-lead ECG showed normal sinus rhythm with no specific ST and T changes.
Supplemental Figure 2: Chest x-ray showed no cardiopulmonary pathology.
