# Supplementary material for: ALCAPA Presents in an Adult with Exercise Intolerance but Preserved Cardiac Function
Source: Case Rep Cardiol. 2012 Aug 29;2012:471759. doi: 10.1155/2012/471759 (PMC4007788; doi:10.1155/2012/471759)
Supplement: Supplementary file 1 — Supplemental Figure 1: 12-lead ECG showed normal sinus rhythm with no specific ST and T changes. Supplemental Figure 2: Chest x-ray showed no cardiopulmonary pathology. [file 471759.f1.pdf]

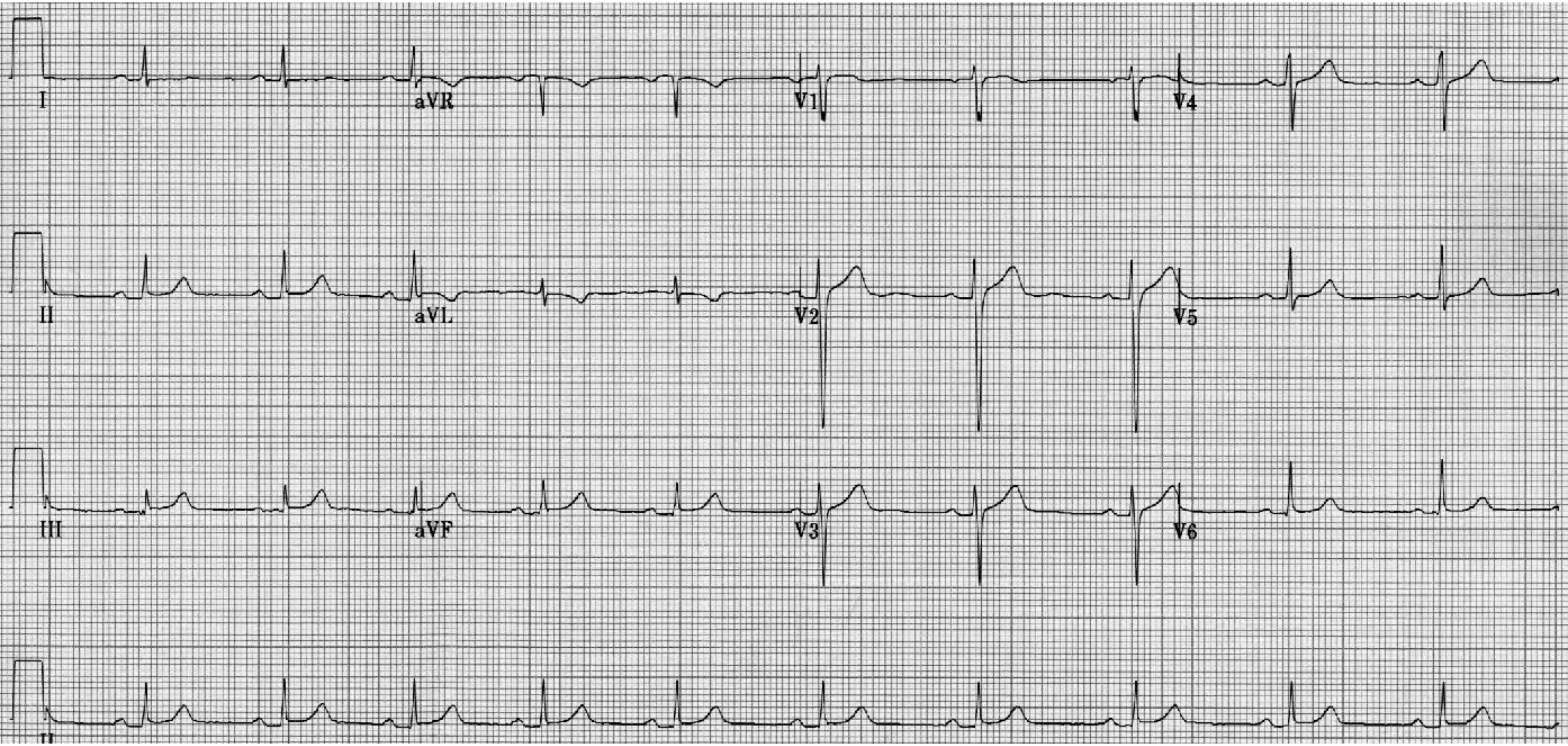


Supplemental Figure 1: 12-lead ECG showed normal sinus rhythm with no specific ST and T changes.

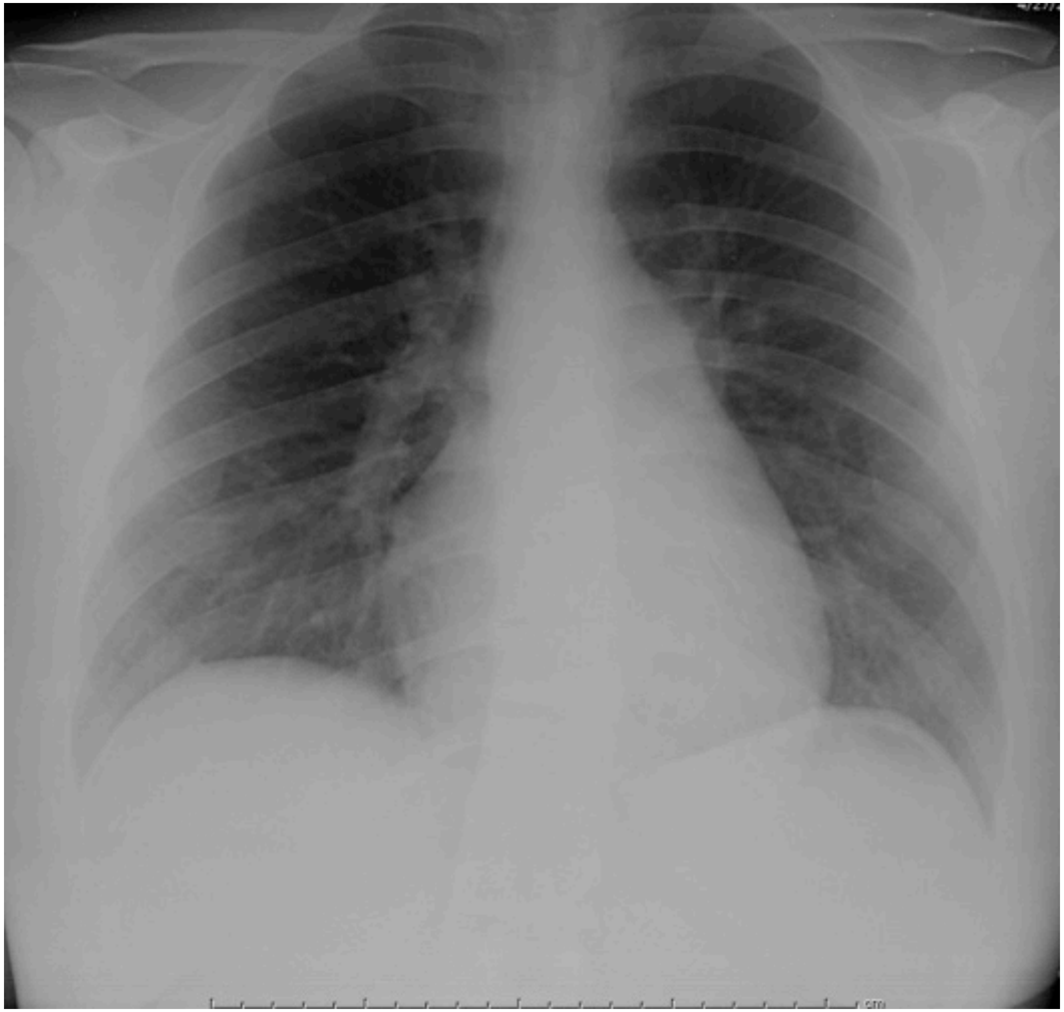

Supplemental Figure 2: Chest x-ray showed no cardiopulmonary pathology
